# With a little help from our friends: strong collaboration and networks for public health

**DOI:** 10.2807/1560-7917.ES.2026.31.2.202601151

**Published:** 2026-01-15

**Authors:** Eurosurveillance editorial team

**Affiliations:** 1 European Centre for Disease Prevention and Control (ECDC), Stockholm, Sweden

**Keywords:** public health, Europe

As we begin a new year, we reflect on events and our initiatives in 2025 and offer a forward-looking perspective of what may come in 2026.

Looking back, just when we thought we had finally overcome the challenges of the COVID-19 pandemic, 2025 presented new public health and societal challenges. Aside from various infectious disease outbreaks and continuously increasing antimicrobial resistance, we have been faced with the growing impact of mis- and disinformation. Amplified by new technologies, tools and actors, these phenomena endanger the mission and vision of evidence-based public health decision making. In this context, continuously providing trusted and well-vetted scientific information is important.

The *Eurosurveillance* annual theme for 2025, Vaccine-preventable diseases in humans – today's challenges and tomorrow's opportunities, was timely. Among the total 217 articles published during the year (131 regular articles, 69 rapid communications, 17 editorials or letters), aspects of vaccination in support of related action and policies were covered in 34 articles. Notably, a cohort study from Denmark showed real-world evidence of stable protection against human papillomavirus (HPV)-related cervical cancer and evidence that vaccines work [1]. In Denmark, cancerogenic HPV16/18 viruses have been almost eliminated among vaccinated women, and prevalence in women who have not been vaccinated against HPV remains stable and low, indicating population immunity. The annual theme was also reflected in our scientific seminar: Vaccines and tomorrow's opportunities for public health held at the European Scientific Conference on Applied Infectious Disease Epidemiology (ESCAIDE) in November 2025. It was well attended and covered both aspects of basic science and important future opportunities for public health.

Fact-checking and quality assurance are complex and time-consuming; both require dedicated experts from a wide range of fields who are ready to collaborate for a common good such as preserving scientific integrity. As *Eurosurveillance* editors, we take pride in working closely with our authors throughout the editorial process to optimise logic and language in their articles so that the reader can follow the line of thought easily and enjoy the reading experience. While supporting tools to speed up quality improvement exist and have grown in numbers, time is still needed for coordination and human oversight of their outputs. The results from an external evaluation of the journal’s activities attest that our contributors and readers value our quality assurance efforts and encouraged us to continue our way of working, while striving to shorten publication turnaround times. Rapid communications were once again confirmed as a strong asset of *Eurosurveillance*.

We recognise our board members who have a formal role as associate editors or editorial advisors, our many other supporters with no formal role, including our colleagues at the European Centre for Disease Prevention and Control (ECDC), and our peer reviewers who are important for our editorial decision making. Our authors and we as editors need your wisdom, constructive critique and encouragement to ensure the information we publish is as rigorous as possible and presented in a clear and transparent manner. We are thankful for your continued support and guidance that help us select interesting, relevant and sound contributions for *Eurosurveillance*. We could not do it without you, and we value your support despite your many other tasks, limited time and often limited institutional acknowledgement. We gratefully acknowledge the 509 reviewers who supported us in 2025 and hope many of you were able to profit from our reviewer recognition schemes. As per our long-standing tradition, we published the names of our peer reviewers in the first issue of 2026 [2]. Our reviewers come from 44 countries on six continents, 264 are male and 244 female (one unknown).

In this editorial, we also renew our thanks to our publisher, ECDC and its Director, who fund *Eurosurveillance* and grant editorial independence to the editor-in-chief and the journal. Continuous funding enables our Diamond Open Access publishing, which has a recognised added value through removing barriers to submitting and accessing content for both authors and readers. We are also grateful to our board members who act as our ambassadors and who support our editorial independence and scientific integrity and who advise us in questions related to publication ethics. We appreciate and value our authors who entrust us by submitting articles on the results of their work to *Eurosurveillance*. Finally, we wish to pay tribute to Professor Angus Nicoll who sadly passed away at the beginning of 2026. He was a free thinker and openminded public health visionary whose support and contributions to *Eurosurveillance* from the start were instrumental for the journal’s successes [3,4,5].

Despite challenges, 2025 was a successful year for the journal. We received more submissions (n = 1,050) than at any time in the past except for the first two pandemic years, plus an increasing number of pre-submission enquiries (n = 149). We accepted one in five articles for publication and, among contributions received from 85 countries, we published articles from 29 countries. While we selected manuscripts with our European focus in mind, we published ─ as in the past ─ several articles with European relevance from countries outside our continent. The citation metrics and ranking remained roughly similar to previous years with *Eurosurveillance* remaining in Q1 throughout and often as one of the top 10 among leading journals in the same category: Clarivate analytics’ Journal Citation Report (impact factor 7.8, #6 Infectious Disease journals), Google scholar metrics (#3 Epidemiology, #9 Communicable Diseases), SCImago journal rank (2.33 # 115/2,524 Medicine (miscellaneous) #4/181 Infectious Diseases Open Access) and Scopus CiteScore (15.5 #15/357 Infectious Diseases). We held editorial workshops for public health experts in six different countries; they were well received and brought us closer to our contributors.

Looking forward, it is difficult to predict what may happen and which infectious diseases will impact public health most in the near future. However, mis- and disinformation are likely to intensify, and artificial intelligence (AI) tools may take over more of our tasks. As already pointed out, we recognise the importance of addressing mis- and disinformation through rigorous and sound information in the articles we publish, and that we should both harvest opportunities and understand risks from AI tools and ensure human oversight. In 2025, we launched a pilot using AI to create visual abstracts for selected articles. We published three already and will continue the pilot in 2026 – your feedback on this initiative is welcome.

At the start of 2026, the year which marks *Eurosurveillance*’s 30^th^ anniversary, we launched a new homepage to provide an easier gateway to information for our readers and to resources for our authors and reviewers, as suggested in the feedback from the external evaluation. This year, we will celebrate ‘*30 years of collaboration and strong networks for public health*’. Throughout 2026, we will highlight partnerships and strong networks in European public health, where we believe our collaboration has made a difference and had public health impact for Europe and even beyond. These include our editorial board with representatives from all European Union and even more European countries, our long-standing collaboration with European field epidemiology training programmes to support capacity building around scientific communication, and our recently launched network of editors of open access journals published by international public health organisations. Moreover, we encourage collaboration among our authors by specially inviting submissions of Euroroundup articles jointly produced by at least four European countries.

In 2026, *Eurosurveillance* will uphold its mission and vision of being an authoritative and representative public health voice of the communicable disease community in Europe and beyond. Together with our contributors, partners and networks, we will continue to provide facts and guidance for health professionals and decision makers that facilitate the implementation of effective prevention and control measures for the benefit of public health.
